# The molecular architecture of dihydropyrindine receptor/L-type Ca^2+^ channel complex

**DOI:** 10.1038/srep08370

**Published:** 2015-02-10

**Authors:** Hongli Hu, Zhao Wang, Risheng Wei, Guizhen Fan, Qiongling Wang, Kaiming Zhang, Chang-Cheng Yin

**Affiliations:** 1 Department of Biophysics, Peking University Health Science Centre, Peking University, 38 College Road, Beijing 100191, China

## Abstract

Dihydropyridine receptor (DHPR), an L-type Ca^2+^ channel complex, plays an essential role in muscle contraction, secretion, integration of synaptic input in neurons and synaptic transmission. The molecular architecture of DHPR complex remains elusive. Here we present a 15-Å resolution cryo-electron microscopy structure of the skeletal DHPR/L-type Ca^2+^ channel complex. The DHPR has an asymmetrical main body joined by a hook-like extension. The main body is composed of a “trapezoid” and a “tetrahedroid”. Homologous crystal structure docking and site-specific antibody labelling revealed that the α1 and α2 subunits are located in the “trapezoid” and the β subunit is located in the “tetrahedroid”. This structure revealed the molecular architecture of a eukaryotic Ca^2+^ channel complex. Furthermore, this structure provides structural insights into the key elements of DHPR involved in physical coupling with the RyR/Ca^2+^ release channel and shed light onto the mechanism of excitation-contraction coupling.

Ion channels are fundamental in living kingdom. Great efforts have been made to solve the structures of ion channels in order to decipher the molecular architecture and action mechanisms of different type of ion channels. Currently, the molecular architectures of K^+^, Na^+^, and Cl^−^ channels have been revealed. However, the molecular architecture of Ca^2+^ channels, which play pivotal roles in a variety of biological processes, such as muscle contraction, secretion, integration of synaptic input in neurons and synaptic transmission, remains elusive. Recently, a bacterial voltage-gated Ca^2+^ channel, Ca_v_Ab, which is a homotetramer composed of a single peptide, was constructed from its Na^+^ channel homologue, Na_v_Ab, and the crystal structure was solved by X-ray crystallography. From which, the Ca^2+^ selectivity and conduction mechanism was proposed^1^. However, the molecular architecture of a eukaryotic, multiple-subunit Ca^2+^ channel complex is still not available.

The skeletal dihydropyridine receptor (DHPR) is an L-type Ca^2+^ channel (Ca_v_1.1). It is a 450 kDa protein complex composed of five subunits (α1, 176 kDa; α2, 147 kDa; δ, 24 kDa; β, 56 kDa; and γ, 34 kDa) in a molar ratio of 1:1:1:1:1 (Ref. 2). The α2/δ are encoded by the same gene and linked by a disulphide bond. The α2/δ functions by enhancing membrane trafficking and increasing current amplitude^3^,^4^. The β subunit is in the cytoplasmic side and affects the channel gating properties and the trafficking of the α1 subunit^4^,^5^. The crystal structure of β subunit reveals that it interacts with α1 through a conserved α1-interaction domain (AID)^6^,^7^. These subunits function as auxiliary for the main part of the channel α1 subunit, which is the voltage sensor and also forms the Ca^2+^ channel^2^.

Structural determination of DHPR/L-type Ca^2+^ channel complex has been hampered since its first purification in 1987 due to the extreme difficulty to obtain chemically pure and physically homogenous protein sample for X-ray crystallography or electron microscopy studies. The structure has been stuck at beyond 20-Å resolution since 1990′s. At such resolution, only the morphology of DHPR is obtained, the ion-conduction channel, the membrane topology, even the location of subunits, remain unresolved. By improving the purification procedure, we made a breakthrough in obtaining chemically pure and physically homogenous DHPR sample, enabling us to break the 20-Å resolution barrier and obtain a higher resolution structure of DHPR/L-type Ca^2+^ channel complex. Here, we present a 15-Å cryo-electron microscopy (cryo-EM) structure of the skeletal DHPR/L-type Ca^2+^ channel complex. Combining with antibody labelling and cryo-EM identification of the location of key subunits, we unambiguously determined the membrane topology and resolved the ion-conduction channel. This structure revealed the molecular architecture of a eukaryotic, multiple-subunit Ca^2+^ channel complex. Furthermore, this structure provides structural insights into the key elements of DHPR involved in physical coupling with the ryanodine receptor (RyR)/Ca^2+^ release channel and shed light onto the mechanism of excitation-contraction coupling (E–C coupling).

## Results and Discussion

### Improvement of DHPR sample for cryo-EM

**Figure 1:**
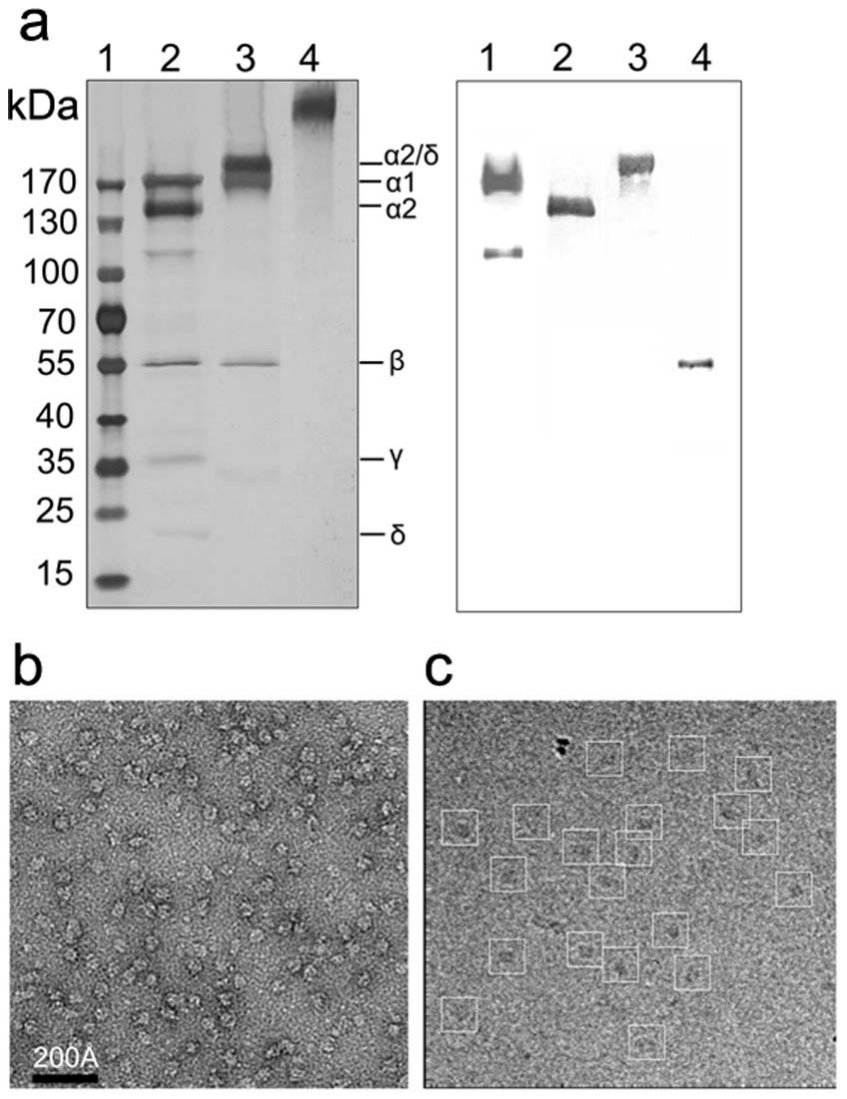
Biochemical characterization and electron microscopy of purified DHPR. (a) Left: Electrophoresis analysis of DHPR. Lane 1, molecular markers; lane 2, DHPR in reducing conditions; lane 3, DHPR in non-reducing conditions; lane 4, DHPR in native conditions. All subunits of DHPR, including the α1 (176 kDa), α2 (147 kDa), β (56 kDa), γ (34 kDa) and δ (20 kDa), exist in the purified DHPR as indicated. Right: Western blotting analysis. lane 1, anti-α1 in reducing conditions; lane 2, anti-α2 in reducing conditions; lane 3, anti-α2 in non-reducing conditions; lane 4, anti-β in reducing conditions. (b) Electron microscopic picture of negatively-stained DHPR sample. (c) Cryo-EM picture of DHPR particles embedded in vitreous ice. Individual DHPR particles are clearly identified and boxed.

Due to the relatively smaller size (450 kDa) and lack of any asymmetry, it would be difficult to obtain high-resolution structure by cryo-EM and single particle analysis if the sample is inhomogeneous. One of the possible reasons that previous structural studies did not obtain a high-resolution structure of DHPR is due to sample heterogeneity. To overcome this difficulty, we first improved DHPR purification procedure (see Methods) and purified DHPR using this new method. The protein purified by our new procedure consists of 5 bands with molecular weights 176-, 147-, 56-, 34- and 24-kDa, respectively, as identified by SDS-PAGE in reducing conditions, which correspond to the α1, α2, β, γ and δ subunits, respectively (Fig. 1a, left panel). In non-reducing conditions, however, the α2 and δ bands vanished; instead, a band slightly above α1 with an apparent molecular weight ~180-kDa appeared which corresponds to the α2/δ complex connected by a disulphide bond. The identities of subunits were confirmed by Western blotting analysis (Fig. 1a, right panel). The chemical purity of the protein sample can be confirmed by the cleanness of the SDS-PAGE gel— apart from the five bands belonging to the DHPR complex, there are no other bands apparently recognizable. Western blot identified the band below α2 being part of α1 (Fig. 1a, right panel, lane 1), presumably resulted from endogenous protease cleavage. Native PAGE gel of the purified protein sample showed only a single band with an apparent molecular weight ~450-kDa (Fig. 1a, left panel, lane 4), indicating the structural integrity of DHPR complex— all the subunits are within a multiple-subunit complex, including the peptide below α2. The chemical purity of the protein sample is further confirmed by this native PAGE gel— apart from the 450-kDa complex band, there are no other bands apparently visible. Negative-staining electron microscopy revealed that the purified DHPR sample contains uniform particles, indicating the DHPR particles are physically homogeneous (Fig. 1b). The chemically pure and physically homogeneous DHPR sample paved the way for high-resolution structural analysis by cryo-EM and single particle analysis.

### The ion-conduction channel

**Figure 2:**
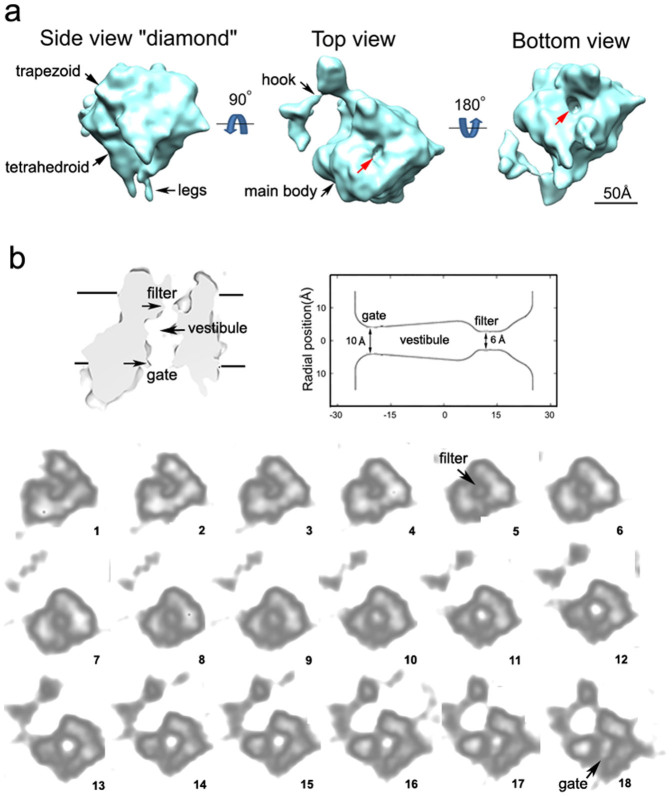
Three-dimensional structure and ion-conduction channel of DHPR complex. (a) Three-dimensional structure. Left, side view (“diamond”), viewed vertical to the pseudo-4-fold axis; Midle, top view, obtained by a down-wards rotation of 90° of the “diamond” view along the horizontal axis; Right, bottom view, obtained by a down-wards rotation of 180° of the top view along the horizontal axis. Structural features (main body, hook, leg, trapezoid, tetrahedroid & central depression) are indicated by arrows. The central depressions (indicated by red arrows) on both sides of the rectangular-shaped main body of DHPR suggest that these could be the openings of the ion-conduction channel. (b) Ion-conduction channel. Upper panel, a cut-through view of the channel in vertical direction. The positions of putative membrane, the ion selectivity filter and the gate are indicated. Lower panel, sections of the channel in horizontal direction. The slices, from slice 1 to slice 18, contain the putative transmembrane region of DHPR, viewed from extracellular side to the cytoplasmic side. The distance between slices is 2 pixels (1.43 Å/pixel); the thickness of each slice is 2 pixels. The putative selectivity filter and the gate are located in slice 5 and slice 18, respectively.

Several groups have performed structural studies of DHPR^8^,^9^,^10^,^11^,^12^,^13^,^14^, and their results have varied; the proposed membrane topology of DHPR is controversial, and the ion-conduction channel in these studies is not resolved due to the low resolution achieved (>20 Å). Using our new purification procedure, we obtained chemically pure and physically homogeneous DHPR sample (Fig. 1). Cryo-EM images of DHPR particles were collected (Fig. 1c) and a total of 25,300 individual DHPR particle images were analysed using single-particle image processing software EMAN 1.8 (Ref. 15), enabling us to break the 20-Å resolution barrier and obtain a structure at 15-Å according to the gold-standard criterion (Supplementary Fig. 1). The final three-dimensional (3D)-EM map is shown in Figure 2a. The map has an asymmetrical main body with dimensions of 90Å × 88Å × 125Å and a hook-like extension. The main body is composed of a “trapezoid” and a “tetrahedroid”, with the “tetrahedroid” attached to the bottom of the “trapezoid”; at the tip region of the “tetrahedroid” there are two “legs”. The map is asymmetric. In one particular side view, it looks like a “diamond” (Fig. 2a, left panel); when viewed from the top and the bottom, it has a ~88 × 88Å rectangular-shaped main body with a hook-like extension attached to one of the corners of the rectangle. In the centre of the rectangle, the top and bottom have a depression with a diameter of ~10 Å and ~14 Å (at the threshold of 1.8, indicated by red arrows) respectively (Fig. 2a, middle and right panels).

The presence of depressions on both sides of the rectangular-shaped main body of DHPR hints that they could be the openings of the ion-conduction channel. To see if this is the case, we analysed the 3D-EM map by sectioning the map along the axis connecting the centres of the top and bottom depressions. We first cut the map in vertical direction (Figure 2b, upper panel). As can be seen from the cut-through view, a channel with characteristics of a Ca^2+^/Na^+^ channel appears^1^,^16^: it has a funnel-shaped opening at the top, then narrows down to a neck with the smallest diameter (~6 Å) of the channel, presumably corresponding to the ion selectivity filter; after that, it runs into a vestibule (central cavity), gradually narrowing down to a narrow opening (~10 Å), presumably corresponding to the gate of the channel. To see these characteristics in detail, we then sectioned the map in horizontal direction using a 2 pixel step (Figure 2b, lower panel). The data show that a central hole runs through all the slices of the “trapezoid” part in the main body, forming a channel. The channel diameter gradually increases from its narrowest point, ~6 Å, in the 5th–6th slices, which presumably corresponds to the selectivity filter, to its widest point in the 12th–13th slice, and then it narrows back down to ~10 Å in the 18th slice, which presumably corresponds to the gate. After that, it runs out of the map. The length of this channel, from the putative selectivity filter to the gate, is ~3.7 nm, long enough to extend across a lipid bilayer. Also, the channel is surrounded by densities displaying psuedo-4-fold symmetry, which is consistent with the model that Ca^2+^ channels are composed of 4 homologous domains surrounding a central channel^2^. We therefore conclude that this channel we observed corresponds to the ion-conduction channel of the DHPR/L-type Ca^2+^ channel complex.

### The membrane topology

**Figure 3:**
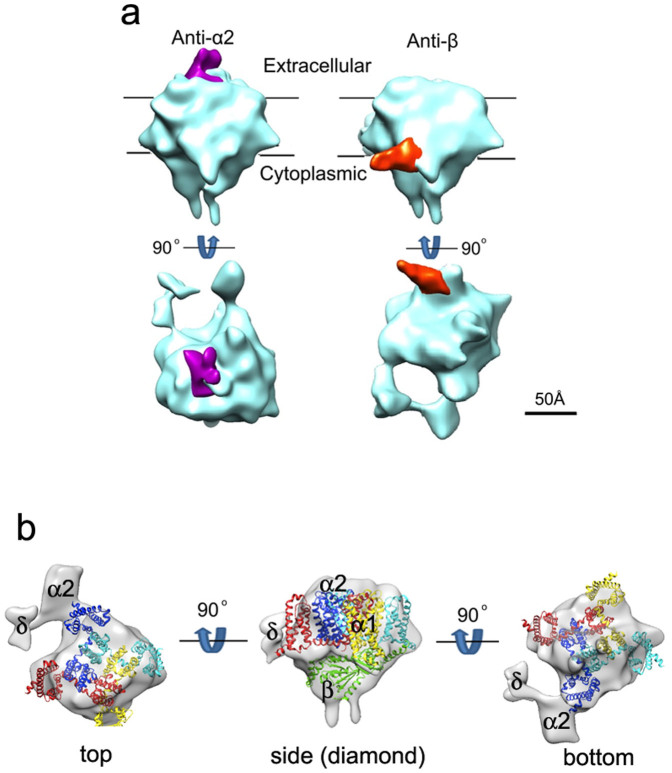
Membrane topology determination, crystal structural docking and subunit assignments of DHPR complex. (a) Membrane topology. The 3D EM maps of DHPR are coloured in cyan, the anti-α2 and anti-β antibodies are coloured in magenta and orange, respectively. The position of putative lipid bilayer is marked according to Figure 2b. As the α2 subunit is located on the extracellular side and the β subunit is located on the cytoplasmic side^17^,^18^, the membrane topology of DHPR can be determined from the subunit-specific antibody labelling as shown here, which is consistent with the direction and orientation of ion-conduction channel shown in Figure 2b. (b) Crystal structure docking and subunit assignments. Crystal structure of Ca_v_Ab (PDB-ID:4MS2), a constructed bacterial Ca^2+^ channel and a homologue of the α1 subunit of DHPR, and the cardiac DHPR β2a (a homologue of skeletal β subunit) complex with AID of the α_1_ subunit (PDB ID:1T0J) were fit into the pseudo-4-fold “trapezoid” and the “tetrahedroid” regions of the DHPR EM-map, respectively. Coloured ribbons represent the atomic models of the I–IV subunits of Ca_v_Ab (red, yellow, cyan and blue) and the DHPR β subunit (green). Left: top view, viewed from the extracellular side, down the putative ion-conduction channel. Middle: side view (“diamond”), viewed parallel to the membrane plane, obtained by a rotation of 90° up-wards of the top view. Right: bottom view, viewed from the cytoplasmic side, obtained by a down-wards rotation of 180° of the top view. The putative locations of subunits α1, α2, β and δ are indicated.

To confirm that the channel we revealed is indeed the actual ion-conduction channel of DHPR, we then determined the membrane topology of the DHPR complex by subunit-specific antibody labelling followed by key subunit localization using cryo-EM and single particle analysis. Previous studies have tried to determine the membrane topology of DHPR and the locations of subunits by antibody and/or YFP-fusion protein or ConA labelling^8^,^11^,^12^,^13^,^14^. In these studies, negative staining electron microscopy instead of cryo-EM, and in most cases, only projection images were used, this could potentially produce artefacts and lead to fault interpretation. To unambiguously determine the locations of subunits and membrane topology, we used cryo-EM and 3D reconstruction. Two monoclonal antibodies were chosen for our purpose— the first, against the α2 subunit (anti-α2) and the second, against the β subunit (anti-β) of DHPR. We incubated antibodies with DHPR in a molar ratio of 10:1 to ensure that all DHPRs would form DHPR-antibody complexes and then collected cryo-EM images. These images were analysed using EMAN1.8, and the final 3D-EM maps are presented in Figure 3a. As shown in this figure, the antibody against the α2 subunit is located on top of the “trapezoid” in the main body, while the antibody against the β subunit is located at the tip region of the “tetrahedroid” in the main body. Previous studies have established that the α2 subunit of DHPR is located on the extracellular side, and the β subunit of DHPR is located on the cytoplasmic side^17^,^18^. This enabled us to determine the membrane topology of the DHPR complex as follows: the top of the “trapezoid” in the main body faces the extracellular side, and the tip region of the “tetrahedroid” in the main body faces the cytoplasmic side. This membrane topology is consistent with the direction and orientation of the ion-conduction channel we showed and demonstrates that this is indeed the actual ion-conduction channel of the DHPR/L-type Ca^2+^ channel.

### The molecular architecture of DHPR/L-type Ca^2+^ channel complex

The determination of membrane topology and the ion-conduction channel of DHPR enabled us to dock homologous crystal structures of recently solved constructed bacterial Ca^2+^ channel, Ca_v_Ab (a homologue of the α1 subunit of DHPR)^1^, and the β subunit of the cardiac DHPR β2a (a homologue of skeletal β subunit) complex with an AID segment of α1 (Ref. 6) into our 3D cryo-EM map (Fig. 3b). We first docked the crystal structure of β2a-AID (PDB-ID 1T0J) into the DHPR cryo-EM map. The crystal structure of the β2a subunit has a tetrahedroid shape, which fits well into the “tetrahedroid” region of the DHPR cryo-EM map (Fig. 3b, middle panel). We then, using the AID as a guide, docked the crystal structure of Ca_v_Ab (PDB-ID 4MS2) (Ref. 1) into the DHPR cryo-EM map, making use of the fact that the AID segment is an extension of the 6th transmembrane helix in repeat I of the DHPR α1 subunit^6^,^7^. As can be seen from Figure 3b, the crystal structures of both the constructed bacterial Ca^2+^ channel and the cardiac DHPR β subunit fit the cryo-EM map quite well. From this docking, we identified the putative positions of the ion selectivity filter, located at its narrowest point (~6 Å), and the gate of the Ca^2+^ channel, located at the end of the ion-conduction channel. The positions of both the ion selectivity filter and the gate are consistent with the sectioning analysis (see Fig. 2b). Based on the positions of these two crystal structures and subunit-specific antibody labelling, we assigned the locations of the DHPR subunits α1, α2, β and δ (Fig. 3b).

**Figure 4:**
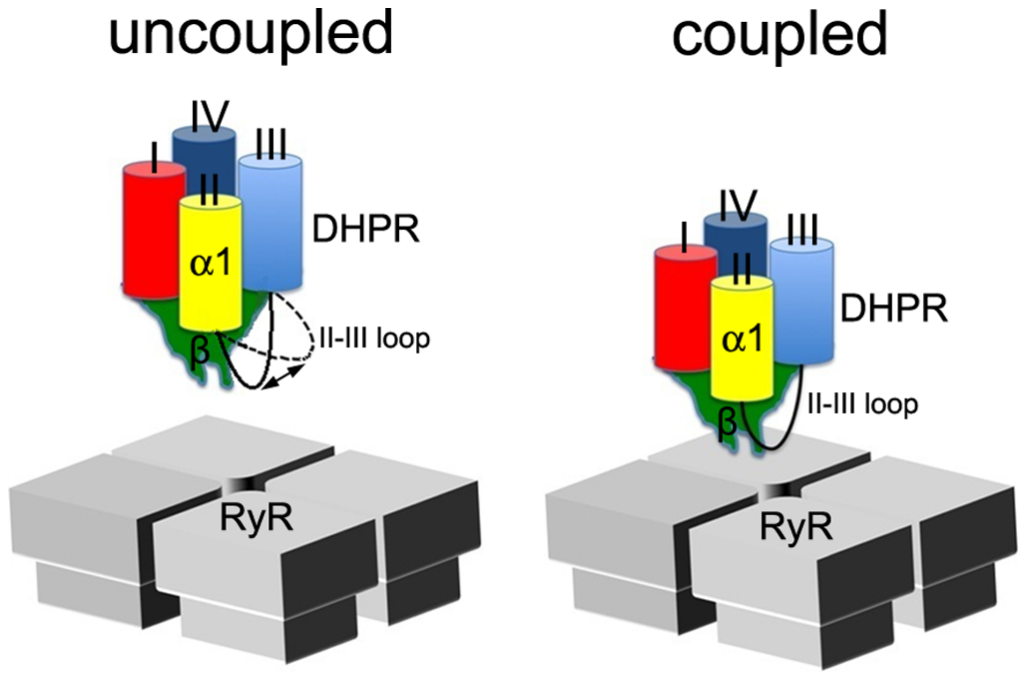
Model of physical coupling between DHPR and RyR1. Due to the mobile nature of the II–III loop of the DHPR α1 subunit, it might be difficult for it to find its coupling site in RyR1 without the interaction of the β subunit of DHPR with RyR1 (left panel); with the docking of the β subunit to RyR1, which might act as an “anchor” and a “fulcrum” of the DHPR complex due to its rigid nature, the II–III loop of DHPR α1 subunit could find and interact with its coupling site in RyR1 and transmit the signal from DHPR to RyR1during excitation-contraction coupling (right panel).

It has been shown that the flexible II–III loop of the α1 subunit is a key element in the physical coupling to the ryanodine receptor (RyR)/Ca^2+^ release channel and E–C coupling^19^,^20^,^21^,^22^,^23^,^24^. It has also been shown that the β subunit interacts with the RyR/Ca^2+^ release channel and is an indispensable element in the formation of DHPR “tetrads”, units of group of 4 DHPR/Ca^2+^ channels and the structural and functional units for E–C coupling in skeletal muscle^25^,^26^,^27^,^28^,^29^. Why the β subunit is essential for E–C coupling is not known. The molecular architecture of DHPR complex provides the structural basis: the tip region of the rigid “tetrahedroid” β subunit, specifically the two leg-like structures, are the most probable sites to interact with the corner region of the square-shaped RyR cytoplasmic region and act as an “anchor” and a “fulcrum” of the DHPR complex, which enables the flexible II–III loop of the α1 subunit to precisely interact with its partner in the same corner region of the square-shaped RyR cytoplasmic assembly, thereby transmitting the signal from DHPR to RyR and vice versa during an E–C coupling process (Fig. 4).

## Methods

### DHPR purification

T-tubule membranes were prepared from rabbit skeletal muscle and homogeneous DHPR/L-type Ca^2+^ channels were purified by two-step chromatography— WGA affinity and DEAE-Sepharose ion exchange as described with modifications^9^. Briefly, T-tubule membranes (3 mg/ml) were solubilized with 1% digitonin in 200 mM NaCl, 10 mM Hepes, pH 7.4, for 45 min, the detergent to protein ratio was 5:1(wt/wt). The insoluble material was removed by centrifugation for 30 min at 120,000 × g. The solubilized membranes were loaded onto a 5-ml WGA fast-flow column specifically designed and custom-made by BIA Seperations (Villach, Austria) and equilibrated with 0.1% digitonin, 200 mM NaCl, 20 mM Hepes, pH 7.4. The detergent concentration in all subsequent buffers was 0.1% digitonin. The unbound proteins were removed by washing with buffer I (0.1% digitonin, 50 mM NaCl, 20 mM Hepes, pH 7.4) for 10 column volumes. The DHPR/L-type Ca^2+^ channels were then eluted with 3 volumes of buffer I with additional 200 mM N-acetylglucosamine (GlcNAc). The GlcNAc-eluted solution was loaded onto a DEAE-Sepharose column equilibrated with buffer I, and the DHPR/L-type Ca^2+^ channels were eluted with a step gradient of NaCl from 100 mM to 300 mM in buffer I. The fractions were analysed by SDS-PAGE and the fractions containing purified proteins were concentrated and mixed with glycerol (final concentration ~25%), quickly frozen in liquid nitrogen and stored at −80°C. All solutions contain protease inhibitor cocktail (Sigma-Aldrich, P8340) and all operations were performed at 4°C.

### SDS-PAGE and Western blotting analysis

Purified protein sample was analysed by 4 ~ 20% linear gradient polyacrylamide gel at reducing and non-reducing (without 2-mercaptoethanol) conditions. Native gel was run in 3 ~ 15% linear gradient polyacrylamide without SDS and 2-mercaptoethanol. Gels were silver stained with 0.1% AgNO_3_.

The proteins separated by 4 ~ 20% SDS-PAGE at reducing and non-reducing conditions were transferred to 0.45-μm polyvinylidene fluoride membranes (Millipore, Billerica, MA) at 400 mA for 2 ~ 4 hr at 4°C. Western blotting analysis was performed with monoclonal antibodies anti-α1 (ab2862, Abcam, Cambridge), anti-α2 (ab2864, Abcam, Cambridge) and anti-β (VD2(1)B12, DHSB, Iowa City, Iowa).

### Cryo-specimen preparation and cryo-EM data collection

The purified DHPRs were diluted in buffer II (200 mM NaCl, 0.05% digitonin, 20 mM Hepes, pH 7.4) to an optimal concentration of ~0.5 mg/ml. DHPR-antibody complexes were prepared by incubating anti-α2 and anti-β antibodies with DHPR at a molar ratio 10:1 at 4°C for at least 1 hour. A 2-μl aliquot of the above-prepared samples was applied on a glow-discharged continuous carbon film coated on a 200-mesh R1.2/1.3 Quantifoil holy grid (Quantifoil Micro Tools GmbH, Jena, Germany). The grid was blotted for 1 ~ 3 seconds and rapidly frozen in liquid ethane using an FEI Vitrobot Mark IV plunge (FEI Company, Hillsboro, Oregon) at 4°C and 100% humidity, and stored in liquid nitrogen prior to data collection. The samples were imaged in an FEI Tecnai F30 field emission gun electron cryo-microscope operated at 300 kV, and image frames were recorded on a Gatan 4k × 4k CCD (Gatan Inc. Pleasanton, CA) at a nominal magnification of 78,000, equivalent to a pixel size of 1.43 Å after calibration. The particle images for the 15-Å resolution map of DHPR were collected from over 600 CCD image frames, and the number of cryo-EM frames recorded for DHPR/anti-α2 and DHPR/anti-β complexes were 95 and 191, respectively, with a defocus range of 2.5 ~ 4 μm. Whole dataset contained 25,300, 18,940 and 23,590 particle images for DHPR, DHPR/anti-α2 and DHPR/anti-β complexes, respectively. Overall ~70% of particle images were used for final map generation.

### Cryo-EM data processing

All the particle images were selected manually using the EMAN2 program *e2boxer.py*. Reference-free 2D averages showed that the DHPRs are conformationally uniform. The contrast transfer function (CTF) parameters of these particle images were manually fitted using the EMAN program *ctfit*. All particles were phase-flipped by *ctfit*, and then an initial model was generated from 2D class averages using EMAN program *startAny*. This model was further refined against the corresponding particle images using the standard EMAN iterative reconstruction algorithm without imposing any symmetry operations.

### Gold-standard eo-test of resolution assessment

The recently developed gold-standard Fourier shell correlation criterion was used to assess the resolution of the final EM-map^30^. The DHPR data were randomly divided into two independent data sets. Initial models were generated by random phasing at 40 Å independently. The two maps produced were aligned by program FOLDERHUNTER before averaging up as a final map. The resolution of 15-Å for the final map was estimated by the 0.143 criterion of FSC curve between map-1 and map-2 without any mask (Supplementary Fig. 1).

### Cryo-EM map post-processing and visualization

“Optimally filtered” map was generated as follows: first, the map was rescaled by a one-dimensional structure factor computed from raw particle images; second, a 15-Å Gauss low-pass filter was applied to the rescaled map. Finial map visualization and rigid-body docking of crystal structures of Ca_V_Ab (PDB-ID:4MS2) and Ca_V_β2a (PDB-ID:1T0J) was performed in Chimera^31^. Comparison between different EM maps was carried out at the same display threshold after map normalization and alignment.

## Author Contributions

C.C.Y. designed research; R.S.W. modified experimental procedures; H.L.H. and R.S.W. prepared sample; H.L.H. and G.Z.F. collected data; Q.L.W. and K.M.Z. did biochemical analysis; Z.W. and C.C.Y. analysed data; C.C.Y., Z.W. and H.L.H. wrote the manuscript with inputs from other authors.

## Supplementary Material

Supplementary Information Supplementary information
