# Supplementary material for: The molecular architecture of dihydropyrindine receptor/L-type Ca2+ channel complex
Source: Sci Rep. 2015 Feb 10;5:8370. doi: 10.1038/srep08370 (PMC4322351; doi:10.1038/srep08370)
Supplement: Supplementary Information [file srep08370-s1.pdf]

The molecular architecture of dihydropyrindine receptor/

L-type Ca^{2+} channel complex

Hongli Hu[1†*], Zhao Wang[1§*], Risheng Wei[1*], Guizhen Fan[1ξ], Qiongling Wang[1¶], Kaiming Zhang[1], Chang-Cheng Yin[1]

[1]Department of Biophysics, Peking University Health Science Centre, Peking University, 38 College Road, Beijing 100191, China

[†]Present address: Wadsworth Centre, New York State Department of Health, Albany, New York 12201, USA.

[§]Present address: National Centre for Macromolecular Imaging, Verna and Marrs McLean Department of Biochemistry and Molecular Biology, Baylor College of Medicine, One Baylor Plaza, Houston, TX 77030, USA

[ξ]Present address: Department of Biochemistry and Molecular Biology, The University of Texas Medical School, Houston, TX 77030, USA

[¶]Present address: Department of Molecular Physiology and Biophysics, Baylor College of Medicine, Houston, TX, 77030, USA

[*]These authors contributed equally to this work

Correspondence should be addressed to Chang-Cheng Yin. Email: ccyin@hsc.pku.edu.cn

SUPPLEMENTARY INFORMATION

Figure S1. Resolution and 3D reconstruction quality assessments of DHPR EM map

(a) Fourier shell correlation (FSC) curve of gold-standard eotest. (b) Representative views of individual DHPR cryo-EM images in different orientations (upper panels) and projections from the final 3D reconstruction in same orientations (lower panels). (c) The angular distribution of particle orientations, showing that almost all orientations are represented in the data set, thereby minimizing any artefacts due to insufficient sampling. (d) Tilt pair validation of 3D reconstruction map according to literature [32].

Figure S2. Section slices of DHPR along the putative ion-conduction channel direction proposed in literature

The slices, from slice 1 to slice 18, represent the putative ion-conduction channel direction of DHPR proposed by Wolf *et al* [11] and others [13,14], viewed from putative extracellular side to the cytoplasmic side. This direction is orthogonal to the ion-conduction channel direction we revealed (ref. Fig. 2b). The distance between slices is 2 pixels (1.43 Å/pixel); the thickness of each slice is 2 pixels. A large niche leading the channel to run side-way into the hydrophobic core of lipid bilayer is illustrated (indicated by arrows).

Figure S3. Comparison of our EM map with the map by Wolf *et al.* (related to Fig. 2)

Our EM map (cyan) is superimposed with the map by Wolf *et al* (grey mesh) [11]. Left:

view as shown in Fig. S2. Middle: view obtained by an anti-clockwise rotation of 90° of the left along the vertical axis. Right: view obtained by a down-wards ration of 90° of the middle along the horizontal axis.

Figure S4. The cryo-EM maps of DHPR displayed at different threshold

When displayed at high threshold, the “hook” shrinks and splits into parts while the main body maintains its shape even at high threshold; when displayed at low threshold, however, the “hook” expands and joins to the main body at two ends and transform into a “handle” (indicated by arrows).

Supplementary Discussion

The ion-conduction channel

The ion-conduction channel proposed in literature [11,13,14] is orthogonal to the ion-conduction channel direction we revealed (ref. Fig. 2b). It appears a “channel” existing in this direction in our EM-map (Suppl. Fig. S2). This “channel”, however, cannot act as the ion-conduction channel for following reasons: first, the densities surrounding the hole do not display psudo-4-fold symmetry, which is contradict to the Ca^{2+} channel model [2]; second, the central hole is not surrounded by densities throughout the channel— it runs sideway in the middle of the map (indicated by arrows in Suppl. Fig. S2), which would run into the hydrophobic core of the lipid bilayer; and third, the density-enclosed channel (from the 1st slice to the 4th slice) is only 1.14 nm, far too short to go across a lipid bilayer.

Comparison of our EM-map with EM-maps in literature

Consistent with previous studies [9,11-14] , our EM map has similar profile with the previously published EM-maps: the DHPR is composed of a main body and a characteristic hook/handle-like density conjoined to the main body. Compared with other maps, the shape of the main body is more close to Serysheva *et al.* [9] (“diamond” vs. “heart”). Using the hook as a register structure, we overlaid our EM map with that of Wolf *et al.* and compared them in detail (Suppl. Fig. S3). As can be seen from this figure, our map can be enclosed by the map of Wolf *et al.* Compared with the map of Wolf *et al.*, the hook region is similar: the length of the hook, the bending angle from the main body and the extending position from the main body matched quite well. The main body region,

however, is smaller than Wolf *et al.* (90Å×88Å×125Å vs. 165Å×145Å×80Å). As the DHPR purified by our new method contained all 5 subunits , which appeared as a single complex with molecular mass ~450kDa (ref. Fig. 1a, lane 2 & lane 4), we believe that the molecular mass in the maps of Wolf *et al.* and others must be larger than 450 kDa therefore contain non-protein mass, most likely detergent and lipids. The difference in detergent and lipid amount is likely caused by different purification methods. In our new method, we washed the column extensively with buffer containing low concentration of detergent before the final elution step and diluted the purified DHPR prior to EM analysis (see Methods), thereby minimised the amount of detergents and lipids attached to the DHPR complex. As the α2 subunit is extra-membrane and connected to the δ subunit just via a disulfide bond, it is mobile. During image processing, the individual particle images were aligned, added together, then averaged to enhance signal-to-noise ratio. Due to the mobile nature, the positions of the α2 subunit in individual particle images would be different from each other. As a result, part of the α2 subunit density would be "averaged out" by image processing. Therefore, it appears smaller than it should be and the majority of the "trapezoid" density in the EM map is contributed mainly by the α1 subunit (MW~176kDa).

Assignments of subunits

Previous studies have assigned the hook region as the α2 subunit of DHPR [11-14]. Based on our structure and membrane topology, however, we believe that the hook region represents part of the α2/δ subunit. As shown in Fig. 3a, the position recognized by the anti-α2 antibody is located on top of α1, which is conjunct to the hook region, suggesting

that the top region above α1 corresponds to part of α2. As the hook region is connected to the region identified as α2, it is reasonable to assume that the hook region is an extension of the α2/δ subunit. The α2 subunit is linked to the δ subunit via a disulphide bond and is proposed to be located in the extracellular side [17]. Currently, there are two models concerning how is the α2/δ subunit associated with the DHPR complex— besides a direct interaction of the α2 subunit with the α1 subunit in the extracellular side, there exists another element that "anchors" the α2 subunit to the plasma membrane: the first model proposed that the δ subunit is a transmembrane protein with a single transmembrane helix, which interacts with the transmembrane domain of the α1 subunit and anchors the α2 subunit to the membrane [33]; the second model proposed that the δ subunit is not transmembrane, but instead extracellular and anchored via a GPI link to the membrane [34]. As our EM structure cannot resolve secondary structures (i.e., alpha helices), we cannot judge which model is correct. However, our EM structure reveals that the δ subunit is not associated with the α1 subunit, this provides a possibility that the interaction of the α2/δ subunit with the DHPR complex could be dynamic, it could associate to/dissociate from the DHPR complex under different conditions, providing a dynamic way of modulation of the DHPR function.

As the α2 subunit is linked to the δ1 subunit via a disulfide bond and is proposed to be located in the extracellular side, this region should be mobile. To test this hypothesis, we examined the density profiles of our EM map by changing display threshold. When displayed at high threshold, the "hook" shrinks and splits into parts while the main body maintains its shape even at high threshold; when displayed at low threshold, however, the "hook" expands and joins to the main body at two ends and transform into a "handle"

(Suppl. Fig. S4). This demonstrates that the hook region is indeed mobile. Serysheva et al. showed that the “handle” (equivalent of the “hook” in our map) is located at the side of the top part of the “heart”-shaped DHPR, whereas in our case the “hook” is attached to the side of the lower part of the “diamond”-shaped DHPR. The position difference of the “hook/handle” in the EM maps from different research groups again demonstrates that this region is mobile. The mobile nature of the α2/δ subunit implies that the interaction of the α2/δ subunit with the DHPR complex could be dynamic; it could associate to/dissociate from the DHPR complex under different conditions, providing a dynamic way of modulation of the DHPR function. The positions of the hook/handle region in the EM maps are probably not its original position as in the membrane-bound state due to this mobility. The original position of the hook/handle region should be addressed by reconstitution of the DHPR into lipid bilayer and investigated by electron tomography or electron crystallography.

References

32. Rosenthal, P.B. & Henderson, R. Optimal determination of particle orientation, absolute hand, and contrast loss in single-particle electron cryomicroscopy. *J. Mol. Biol.* **333**, 721-745 (2003).

33. Gurnett, C.A., De Waard, M., Campbell, K.P. Dual function of the voltage-dependent Ca^{2+} channel alpha 2 delta subunit in current stimulation and subunit interaction. *Neuron* **16**, 431-440 (1996).

34. Davies, A. et al. The alpha2 delta subunits of voltage-gated calcium channels form GPI-anchored proteins, a posttranslational modification essential for function. *Proc. Natl. Acad. Sci. USA* **107**, 1654-1659 (2010).

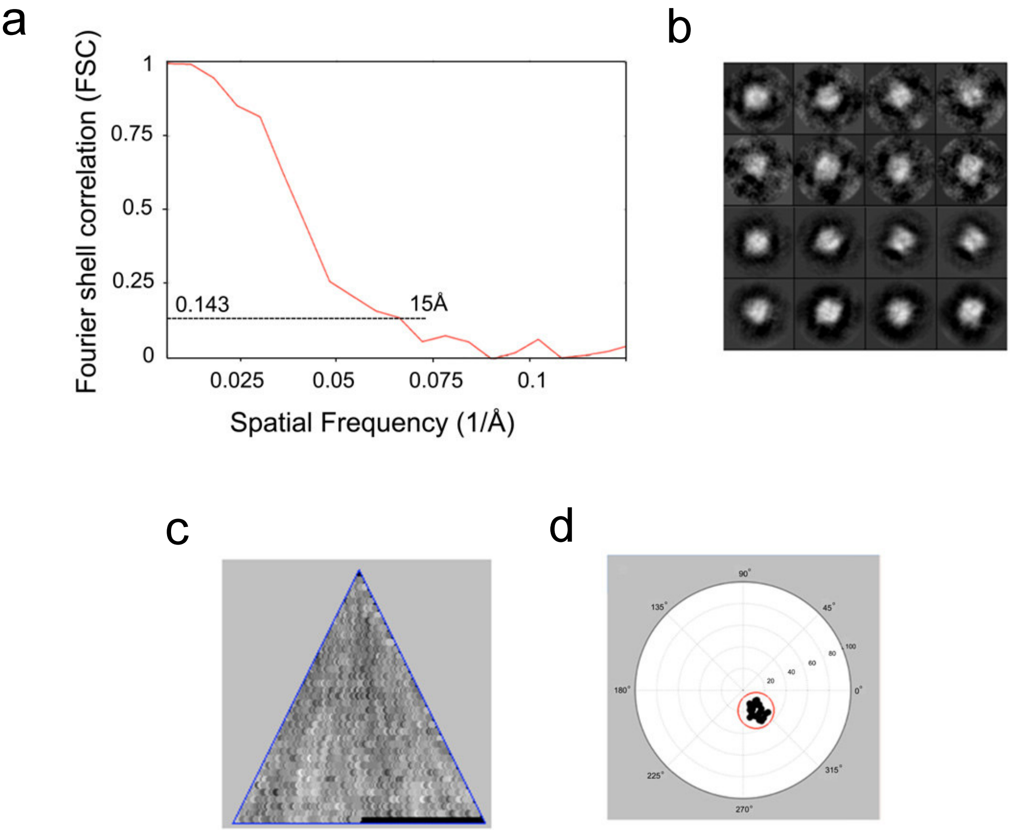
a
Fourier shell correlation (FSC)
1
0.75
0.5
0.25
0
0.143
15Å
0.025
0.05
0.075
0.1
Spatial Frequency (1/Å)
b
c
d
90°
135°
45°
100
80
60
40
20
180°
0°
225°
315°
270°

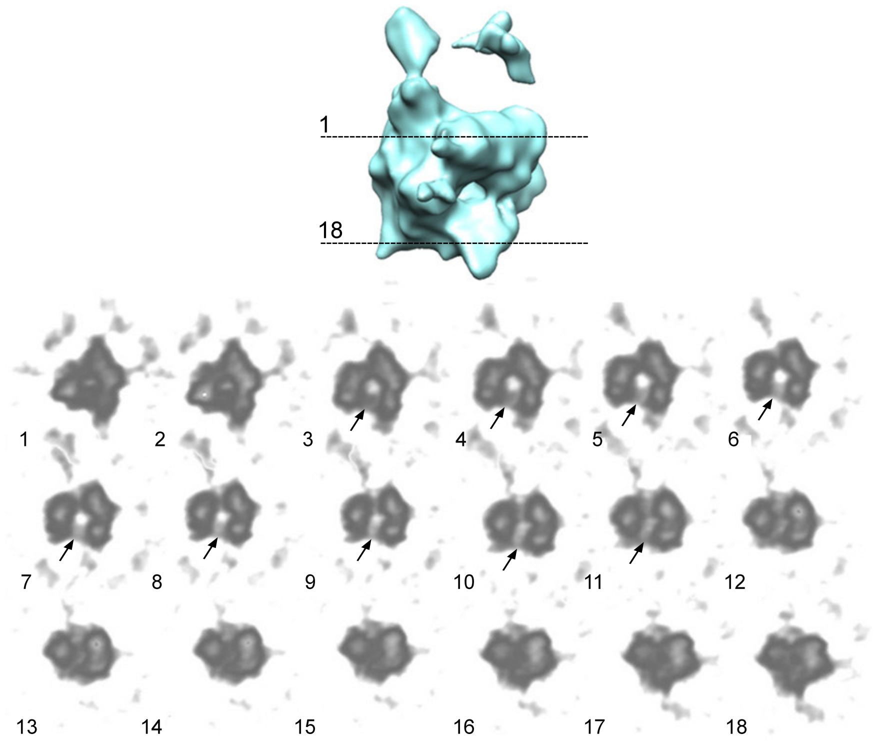
1
18
1
2
3
4
5
6
7
8
9
10
11
12
13
14
15
16
17
18

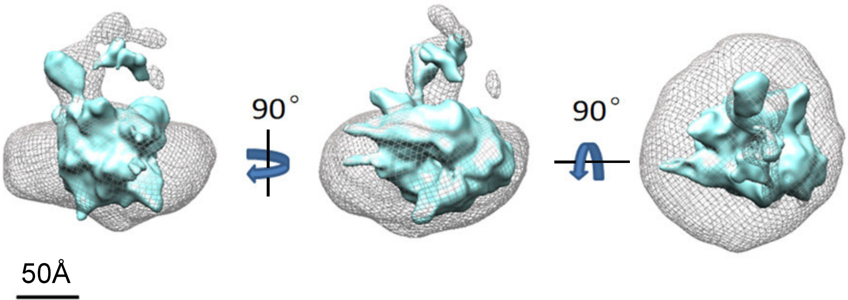
90°
90°
50Å

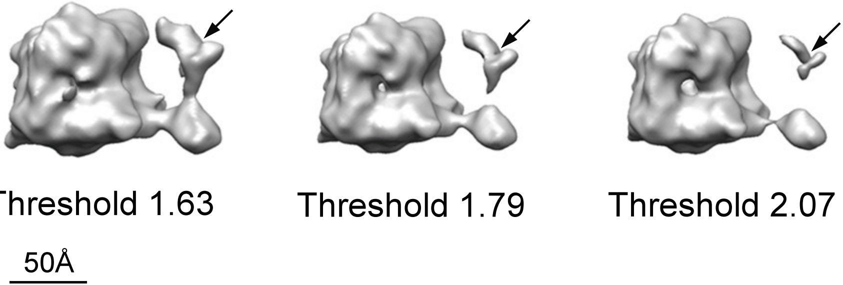
Threshold 1.63
Threshold 1.79
Threshold 2.07
50Å
